# MicroRNAs, Parkinson’s Disease, and Diabetes Mellitus

**DOI:** 10.3390/ijms22062953

**Published:** 2021-03-14

**Authors:** Hsiuying Wang

**Affiliations:** Institute of Statistics, National Yang Ming Chiao Tung University, Hsinchu 30010, Taiwan; wang@stat.nctu.edu.tw

**Keywords:** biomarker, microRNA, Parkinson’s disease, diabetes mellitus

## Abstract

Parkinson’s disease (PD) is a neurodegenerative disorder that affects 1% of the population over the age of 60. Diabetes Mellitus (DM) is a metabolic disorder that affects approximately 25% of adults over the age of 60. Recent studies showed that DM increases the risk of developing PD. The link between DM and PD has been discussed in the literature in relation to different mechanisms including mitochondrial dysfunction, oxidative stress, and protein aggregation. In this paper, we review the common microRNA (miRNA) biomarkers of both diseases. miRNAs play an important role in cell differentiation, development, the regulation of the cell cycle, and apoptosis. They are also involved in the pathology of many diseases. miRNAs can mediate the insulin pathway and glucose absorption. miRNAs can also regulate PD-related genes. Therefore, exploring the common miRNA biomarkers of both PD and DM can shed a light on how these two diseases are correlated, and targeting miRNAs is a potential therapeutic opportunity for both diseases.

## 1. Introduction

Parkinson’s disease (PD) is a chronic neurodegenerative disease that has an increasing prevalence with age [1]. PD affects 1% of the population above 60 years and is called early-onset PD if it begins before age 50. The non-motor symptoms of PD include sleep disorders, depression, cognitive changes, illusions, and delusions [2]. The motor symptoms of PD include tremor, slowed movement, rigid muscles, impaired posture and balance, speech changes, and writing changes [3]. Increasing evidence shows that biological sex is an important factor in the development of PD. The relationship between estrogen exposure and PD risk was investigated, and women with higher cumulative estrogen exposure had a significantly reduced PD risk [4,5].

Several factors can modify the risk of developing PD. The increasing risk factors include pesticides, consumption of dairy products, history of melanoma, and traumatic brain injury, whereas the decreasing risk factors include smoking, caffeine consumption, higher serum urate concentrations, physical activity, and use of ibuprofen and other common medications [6].

1-Methyl-4-phenyl-1,2,3,6-tetrahydropyridine (MPTP) is a drug that can cause irreversible parkinsonism [7]. In addition, metabolic syndrome may be a risk factor for PD development [8,9]. The stimulation of oxidative stress is pivotal to the evolution of metabolic syndrome and PD [10,11]. Besides, a recent study showed the potential link between gut microbiota and PD [12]. It has been proposed that the neurodegenerative cascade may be initiated in the gut with subsequent spreading to the brain and that gut microbiota could be involved in this process.

In recent years, an emerging body of evidence has shown the association between PD and diabetes mellitus (DM). The cause of DM is a result of either the pancreas not producing enough insulin or the body not responding appropriately to insulin. Hyperglycemia affects people who have DM, and chronic hyperglycemia is associated with long-term damage and dysfunction of different organs [13]. According to the diabetes website (https://www.who.int/health-topics/diabetes#tab=tab_1) of the World Health Organization on 1 March 2021, about 422 million people worldwide have DM in 2021. Both the number of cases and the prevalence of DM have been increasing over the past few decades.

There are two main types of DM: type 1 and type 2. In type 1 DM, the pancreas fails to produce enough insulin. People with type 1 DM must use insulin injections to control their blood glucose. In type 2 DM, the pancreas produces insulin, but the body does not respond appropriately to insulin.

Most people with type 2 DM are obese, and obesity may cause insulin resistance [13]. Patients with DM may have many complications, including retinopathy, nephropathy, peripheral neuropathy, and autonomic neuropathy. There is also an increased incidence of hypertension, atherosclerotic cardiovascular complications, peripheral arterial complications, and abnormalities of lipoprotein metabolism. Diabetic retinopathy (DR) is a common complication of DM, and diabetic kidney disease (DKD) or diabetic nephropathy is a type of chronic kidney disease caused by DM. DKD was reported in approximately 40% of DM patients, and the majority of DKD patients die from cardiovascular diseases and infections [14].

The diagnosis of DM is based on fasting sugar blood tests or A1c blood tests. Compared with the simplicity of DM diagnosis, it is more difficult to conclusively diagnose PD due to the lack of a reference standard test [15]. The diagnosis of PD is based on a review of patients’ signs and symptoms, and neurological and physical examinations. The genetic factor can be identified in 5–10% of the patients. Studies show that PD is associated with five genes: α-synuclein (SNCA); parkin (PARK2); PTEN-induced putative kinase 1 (PINK1); DJ-1 (PARK7); and Leucine-rich repeat kinase 2 (LRRK2) [16,17,18,19]. In addition, a meta-analysis on genome-wide association studies (GWAS) from 13,708 cases and 95,282 controls has identified 28 independent risk alleles at 24 gene loci associated with a risk for PD [20]. The gene expression differences between PD and healthy controls can be used as a potential prognosis of PD. In addition to the gene biomarker, the circulating microRNA (miRNA) can be a useful biomarker for PD as well as DM. In this study, we review the common miRNA biomarkers of PD and DM and discuss the association between PD and DM based on their miRNA biomarkers.

## 2. MicroRNA

miRNA is a small, non-coding RNA about 21–24 nucleotides in length that has important functions in cell differentiation, development, the regulation of the cell cycle, and apoptosis. The first miRNA was discovered in the early 1990s when studying the nematode *Caenorhabditis elegans* regarding the gene lin-14 [21]. miRNAs play an important epigenetic role involved in many diseases and can be overexpressed or repressed in different diseases. The inhibition or replacement of miRNAs is a promising area of study for therapeutics [22].

The biogenesis of miRNA is classified into canonical and non-canonical pathways. Most miRNAs are transcribed from DNA sequences into primary miRNAs (pri-miRNAs) and processed into precursor miRNAs (pre-miRNAs) and mature miRNAs. miRNAs are synthesized from primary miRNAs in two stages by the action of two RNase III-type proteins [23,24]. miRNAs may regulate up to 30% of the protein-coding genes in the human genome [25] and are well known to be involved in the initiation and progression of cancers [26,27,28,29,30,31,32,33]. In addition to being tumor suppressors or oncogenes of cancer, miRNAs also contribute to neurological diseases. Let-7b is a miRNA biomarker for anti-NMDA receptor encephalitis [34,35,36]. For neurological diseases, miRNAs were identified to account for PD, amyotrophic lateral sclerosis, frontotemporal dementia, Alzheimer’s disease, spinal muscular atrophy, Prader–Willi syndrome, Niemann–Pick disease, neurofibromatosis, narcolepsy, Friedreich’s ataxia, and ataxia-telangiectasia [36,37,38,39,40,41]. miRNAs are also explored as being related to DM [42].

## 3. MicroRNA Biomarkers

I collected some common miRNA biomarkers of PD and DM from the literature to discuss the association between both diseases. The common biomarkers are listed in Table 1.

**Table 1:** Common microRNAs (miRNAs) related to Parkinson’s disease (PD) and diabetes mellitus (DM).

microRNA	Parkinson’s Disease Reference	Diabetes Reference
miR-92a	[43,44]	[45,46]
miR-100	[43,47]	[48,49]
miR-23a	[43,50]	[51,52]
let-7	[38]	[53]
miR-485	[38,54]	[55]
miR-26	[38,56]	[57,58,59]
miR-146a	[60,61]	[48,62,63,64,65]
miR-335-3p	[60]	[66,67]
miR-155	[54,60,61]	[65,68,69]
miR-1	[56,70]	[71,72,73]
miR-19b-3p	[56,70,74]	[75,76,77]
miR-153	[70]	[76,78]
miR-409-3p	[70]	[79,80]
miR-10a-5p	[70]	[81]
let-7g-3p	[70]	[53,82]
miR-103a-3p	[56,83,84]	[65]
miR-200	[44,54,85,86]	[65]
miR-204	[54,85]	[87,88,89]
miR-21	[54,90,91]	[48,65,92,93,94,95,96,97,98]
miR-96	[99,100]	[101,102]
miR-17	[44,103,104]	[105]
miR-365	[56]	[82,106,107,108]
miR-18a	[44]	[51,82,109,110,111]
miR-125a	[112]	[113,114]
miR-125b	[115]	[106,116,117,118,119]
miR-10b	[112,120]	[121,122]
miR-200c	[44,123]	[106,124]
miR-210	[125]	[48,65,126]
miR-218	[56,127]	[128,129,130,131]
miR-195	[54]	[132,133,134]
miR-7	[54,56]	[135,136]
miR-148a	[54]	[48,51,65,96,111]
miR-182	[137]	[138,139,140]
miR-34a	[141,142]	[63,73,117]
miR-133b	[143]	[71,144]
miR-145	[54,145]	[146,147]
miR-143	[56]	[147,148]
miR-342	[149]	[48,51,65]
miR-26b	[54,150]	[97,151,152]
miR-135b	[54,153]	[154]
miR-22	[50,56]	[155,156,157]
miR-20a	[44,56]	[158,159]
miR-766	[160]	[122]
miR-30b	[56,70,84]	[98]
miR-30c	[56,161,162]	[122,126,163]
miR-148b	[56,162]	[65,152,164]
miR-29a	[38,56,84,149,165]	[166,167]
miR-29c	[165]	[167]
miR-1249	[56,168]	[82,169]
miR-18b	[56,170]	[51,171]
miR-15a	[56]	[172,173]
miR-30a	[38,56]	[126,140,174,175]
miR-9	[56,142,149]	[176]
miR-132	[56]	[177,178]
miR-423	[54,56]	[179,180]
miR-486	[56]	[181,182]
miR-1260	[56]	[183,184]

These two diseases share more miRNA biomarkers than those listed in Table 1. In this paper, we review miRNAs listed in Table 1 to connect PD and DM. The miR-92a, miR-100, and miR-23a were shown to significantly target 244 gene biomarkers of PD that were identified by integrating three datasets including 35 normal control and 25 PD patients’ substantia nigra mRNA expression profiles [43]. The tripartite regulatory network identified miR-18a, -92a, -200a, -200c, -17, and -20a as hub miRNAs that can be considered as possible biomarkers for PD [44]. miR-92a may serve to correct diabetes-associated inflammation and restore normal circadian function in CD34+ cells [45]. miR-100-5p, miR-146a-5p, miR-148a-3p, miR-210-5p, and miR-342-3p were dysregulated in type 1 DM patients compared to controls [65]. In comparison with either normal glucose tolerance or type 2 DM subjects, miR-18a, miR-18b, and miR-23a decreased in impaired glucose tolerance subjects [51]. miR-30, miR-29, let-7, miR-485, and miR-26 were shown to be implicated in PD pathogenesis [38]. Let-7 could be involved in regulating neuronal degeneration in PD and let-7g-3p was up-regulated in the CSF of PD patients [38]. Let-7 regulated multiple aspects of glucose metabolism, and anti-miR-induced let-7 knockdown was suggested as a potential treatment for type 2 DM [53]. Microarray analysis of PD substantia nigra samples revealed that miR-485-5p and miR-204-5p were up-regulated and miR-155-5p and miR-423 were down-regulated. In addition, miR-200, miR-21, miR-195, miR-7, miR-148a, miR-145, miR-26b, and miR-135b also have different expression in PD samples compared to control samples [54]. Overexpression of miR-485 suppressed high glucose-induced proliferation of human mesangial cells [55]. miR-26a in β cells alleviated obesity-induced insulin resistance and hyperinsulinemia, and prevented hyperinsulinemia through targeting several critical regulators of insulin secretion and β cell proliferation [58]. A systematic review of literature summarized miRNAs as differing significantly between individuals with PD and healthy controls and/or between treated and untreated patients with PD including down-regulated miRNAs, miR-30b, miR-30c, miR-26a, miR-148b, miR-1, miR-22, miR-29a, miR-103a-3p, miR-1249, miR-20a, miR-18b, miR-15a, miR-143, miR-19b, and up-regulated miRNAs, miR-30a, miR-7, miR-9, miR-132, miR-423, miR-365, miR-486, miR-1260, and miR-218 [56]. miR-26a could ameliorate bone-specific insulin resistance and bone quality in diabetic mice, which depend on the insulin receptors on osteoblasts [57].

miR-146a, miR-335-3p, and miR-335-5p were down-regulated in idiopathic PD patients and patients with a mutation in the LRRK2 gene versus controls [60]. miR-146a-5p was down-regulated in recently diagnosed type 1 DM patients [65]. miR-21-5p, miR-100-5p, miR-148a, miR-146a-5p, miR-210-5p, and miR-342-3p were dysregulated in type 1 DM patients compared to controls [48]. The expression of miR-335 was negatively correlated with the secretion index in human islets of individuals with prediabetes [66]. An animal study explored the potential involvement of miR-155 in the pathogenesis of diabetes complications [68]. Except for the liver, the miR-155 expression level was significantly decreased in the diabetic kidney, heart, aorta, peripheral blood mononuclear cells, and the sciatic nerve versus the controls. miR-1 and miR-19b-3p showed decreased expression in PD, whereas miR-153, miR-409-3p, miR-10a-5p, and let-7g-3p were found to be up-regulated [70]. Type 2 DM patients expressed decreased levels of miR-1-3p and miR-34a-5p compared with controls [73]. miR-19b targets PD-related genes [74]. The long non-coding RNA maternally expressed gene 3 (MEG3) inhibited high glucose-induced apoptosis and inflammation by regulating the miR-19b/SOCS6 axis through the JAK2/STAT3 signaling pathway in the human retinal microvascular endothelial cells [185]. The miR-153 expression level was increased in IL-1β-treated β cells and primary islets from the diabetic rodents [76]. An insulin resistance group presented a remarkably higher serum miR-409-5p level than a non-insulin resistance group [79]. Acarbose, an α-glucosidase inhibitor, can regulate glucose metabolism through the MAPK pathway and can suppress proinflammatory cytokines by increasing miR-10a-5p and miR-664 in the ileum. Acarbose reduced blood glucose by activating miR-10a-5p in diabetic rats [81]. Let-7g was differentially expressed in patients with or at risk of for type 1 DM [82]. Significant overexpression of miR-103a-3p, miR-30b-5p, and miR-29a-3p was observed in treated patients with PD [84]. miR-21-5p, miR-103a-3p, miR-148b-3p, miR-155-5p, miR-200a-3p, and miR-210-3p were up-regulated in recently diagnosed type 1 DM patients compared with controls [65]. Serum miR-204 was elevated in children and adults with type DM [87].

miR-96-5p was involved in oxidative stress in PD [100]. The overexpression of miR-96 was found to lead to an impairment of insulin signaling and glycogen synthesis in hepatocytes [101]. A significant decrease in miR-17-3p in diabetic retinopathy as well as in proliferative diabetic retinopathy patients was shown when compared with non-diabetic retinopathy patients [105]. miR-125b-5p and miR-365a-3p have strong positive correlations with HbA1c [106]. A study proposed miRNA biomarker panels that efficiently distinguish early-stage PD patients from controls and miR-125a-5p and miR-10b-5p were identified in these miRNA panels [112]. miR-125a is overexpressed in insulin target tissues in a spontaneous rat model of type 2 DM [114]. miR-125b-5p is a putative target gene of the long non-coding RNA brain-derived neurotrophic factor anti-sense that might act as a potential therapeutic target for PD [115]. A titer of islet autoantibodies IAA was negatively associated with miR-10b-5p [122]. miR-200c-3p was down-regulated in the echinomycin-treated PD cellular model [123]. miR-200c-3p was positively correlated with HbA1c [106]. miR-210 target genes were identified to have a significant age-related neurodegenerative disease pathway enrichment including Huntington’s disease, Alzheimer’s disease, and PD [125]. Plasma miR-210 was significantly up-regulated in type 2 DM subjects in contrast to controls [126]. The down-regulation of miR-218 in the brain was related to PD via activation of NF-κB signaling [127]. Glucose up-regulated miR-218 expression, and miR-218 and RUNX2 might be vital targets for use in diagnosing and treating DR [129]. miR-195 was up-regulated in the frontal cortex region of the PD brain [54]. miR-195-5p expression was significantly increased in serum samples from gestational DM patients as compared with those in healthy pregnancies [133]. Serum miR-7 was significantly elevated in the type 2 DM patients and the type 2 DM-associated microvascular complications patients when compared with the controls [135]. Levels of miR-148a-3p were associated with glycemic status and glucose levels [111]. miR-182-5p mediates nigrostriatal protection in the MPTP model of PD [137]. miR-182-5p was very highly expressed in individuals with prediabetes or type 2 DM, and miR-182-5p was observed to be significantly under-expressed in type 2 DM relative to prediabetes [140].

miR-34a was differentially expressed in 1-methyl-4-phenylpyridinium (MPP)+-treated differentiated PC12 cells as a model of PD [142]. miR-34a and miR-9 were up-regulated in MPP+-treated differentiated PC12 cells as a model of PD [142]. miR-34a was increased in type 2 DM patients who were overweight and obese, and miR-34a was differentially affected by glycemia, obesity, insulin treatment, and the presence of nephropathy and diabetic foot [63]. The plasma level of miR-133b was reduced in PD patients compared with the controls [143]. Myocardial-specific miR-133b was confirmed to be down-regulated in diabetic rat hearts [71]. By comparing the miRNAs identified in this experiment with those previously reported to be associated with DKD, miR-133b was up-regulated in urinary exosomes in patients with type 2 DKD [144]. miR-145-3p in the PD group was higher than that in the control group [145]. In rats with type 1 DM, the therapeutic effects of stroke treatment were compared between bone-marrow stromal cells (BMSCs) derived from type 1 DM rats (DM-BMSCs) and BMSCs derived from normal rats (Nor-BMSCs). In vivo, compared with Nor-BMSC or phosphate-buffered saline treatment, DM-BMSC treatment improved functional outcome, decreased serum miR-145 expression, and increased expression of the miR-145 target genes ABCA1 and IGFR1 [146]. miR-342-3p, miR-29a-5p, and miR-9-5p were identified to regulate genes associated with PD such as CTSB and SPPL2B [149]. The level of miR-26b targeting hsc70 was significantly increased in PD substantia nigra pars compacta relative to actin mRNA levels [150]. miR-26b-5p was found to be significantly down-regulated following metformin treatments in patients with type 2 DM [97]. Ectosomes (Ects) are a subpopulation of extracellular vesicles, and the level of miR-26b-5p was significantly different between Ects obtained from patients with type 2 DM and those obtained from healthy controls [151]. miR-26b was detected in the blood of type 2 DM samples that indicated miR-26b as a promising biomarker of type 2 DM [152].

Both in vitro and in vivo, the expression of miR-135b decreased in retinal cells under hyperglycemia exposure and increased in the DM retina [154].

The dysregulations of miR-22 and miR-23a were shown in the comparison between PD patients and control individuals [50]. Three experimental rat groups were analyzed in a study: rats receiving a standard diet (N), rats receiving a high-fat diet (HFD), and rats receiving a high-fat diet (HFD) with simultaneous administration of T2 (HFD-T2). An approximate 50% decrease in the level of miR-22-3p was detected in the serum of HFD-T2 rats in comparison to HFD rats [155]. miR-20a-5p was significantly decreased in women with gestational DM compared with controls [159]. miR-766 could target the gene GBA, and mutations of GBA were the most common genetic risk factor for PD [160]. The blood glucose concentration measured at 120 min of an oral glucose tolerance test was correlated negatively to miR-766-3p [122]. miR-30b-5p was differentially expressed in PD [70]. mRNA and protein profiling of extracellular vesicles extracted from diabetic subjects with the DR group or without the DR group and healthy controls were performed. Modulation of miR-30b-5p inside microvascular cells confirmed their involvement in abnormal angiogenesis [98]. Serum miR-30c-5p levels correlate with disease duration in both multiple system atrophy and PD [161].

miR-30c and miR-148b were down-regulated in PD [162]. miR-30c reduced plasma cholesterol in several diet-induced and diabetic hypercholesterolemic mice [163]. The levels of HbA1c were negatively associated with miR-30c-5p [122]. miR-148b was detected in the blood of type 2 DM samples indicating miR-148b as a promising biomarker of type 2 DM [152]. The level of miR-148b was detected in the sera of healthy controls, individuals with impaired glucose regulation, and type 2 DM patients by real-time polymerase chain reaction (PCR). Compared with those in the healthy control group, the miR-148b level in the impaired glucose regulation group was significantly higher. miR-148b was also significantly higher in the type 2 DM group compared with the other groups [164]. The expression of serum miR-29a and miR-29c expression tended to decrease with PD severity [165]. In the liver, both miR-29a and miR-29c were important negative regulators of insulin signaling via phosphatidylinositol 3-kinase regulation [166]. Evidence showed that miR-29a and miR-29c were increased in skeletal muscle from patients with type 2 DM [167]. miR-1249 was altered in PD with a focus on early-onset PD and late-onset PD patients [168]. A set of miRNAs including miR-18b and miR-1249 inverted their trend after deep brain stimulation treatment, becoming down-regulated compared to PD untreated patients’ samples [170]. miR-1249 was differentially expressed in pre-DM, obese, and non-diabetic individuals at follow-up [82]. miR-1249 was associated with the DM complication nephropathy [169]. The long non-coding RNA KCNQ1OT1 that regulated miR-18b-5p could affect cell proliferation, apoptosis, and fibrosis in diabetic nephropathy [171]. The peripheral blood miR-15a expression levels were significantly decreased in patients with type 2 DM and pre-diabetes individuals exhibiting impaired fasting glucose and impaired glucose tolerance individuals, compared with healthy control subjects [172]. Expression of miR-15a was increased in skeletal muscle obtained from the gestational DM group and type 1 DM group compared with a control group of offspring from the background population [173].

An up-regulation trend was observed for miR-30a-5p in L-dopa-treated PD patients [175]. miR-30a-5p and miR-30c-5p were found to be involved in blood coagulation, platelet activation, glucose metabolism, insulin signaling, and inflammation and were significantly up-regulated in type 2 DM [126]. miR-30a-5p is associated with dysglycaemia and could potentially predict prediabetes [140]. Deregulated plasma levels of miR-30a-5p were observed years before the onset of type 2 DM and pre-DM and could be used to evaluate the risk of developing DM [174]. miR-9-5p was one of several miRNAs that might target 13 genes associated with PD [149]. Experiments based on islet cell lines indicated that the overexpression of miR-9 decreased glucose-stimulated insulin secretion, while the knockdown of miR-9 promotes insulin secretion to a certain degree [176]. Expression of miR-132 was decreased in serum and placenta tissues in gestational DM patients compared with the healthy women [177]. miR-132 played a critical role in the regeneration of mouse islet β cells through the down-regulation of its target Pten, and the miR-132/Pten/Akt/Foxo3 signaling pathway might represent a suitable target to enhance β cell mass [178]. Lowered miR-423 levels in DM patients showed a correlation with vascular endothelial growth factor and an inverse correlation between nitric oxide and endothelial nitric oxide synthase expression. Hence, miR-423 may be involved in the regulation of diabetic vascular retinal proliferation [179]. miR-486-5p identified responders to thiazolidinedione therapy among the insulin-resistant group [181]. The serum levels of miR-486 were significantly reduced in patients with DKD when compared with the healthy control and type 2 DM groups [182]. In the comparison of type 1 DM versus type 2 DM, miR-1260 was differently expressed in the two groups [183]. Circulating miR-1260a was differently expressed at two time points in elderly type 2 DM patients who did not respond to sitagliptin treatment [184].

**Figure 1:**
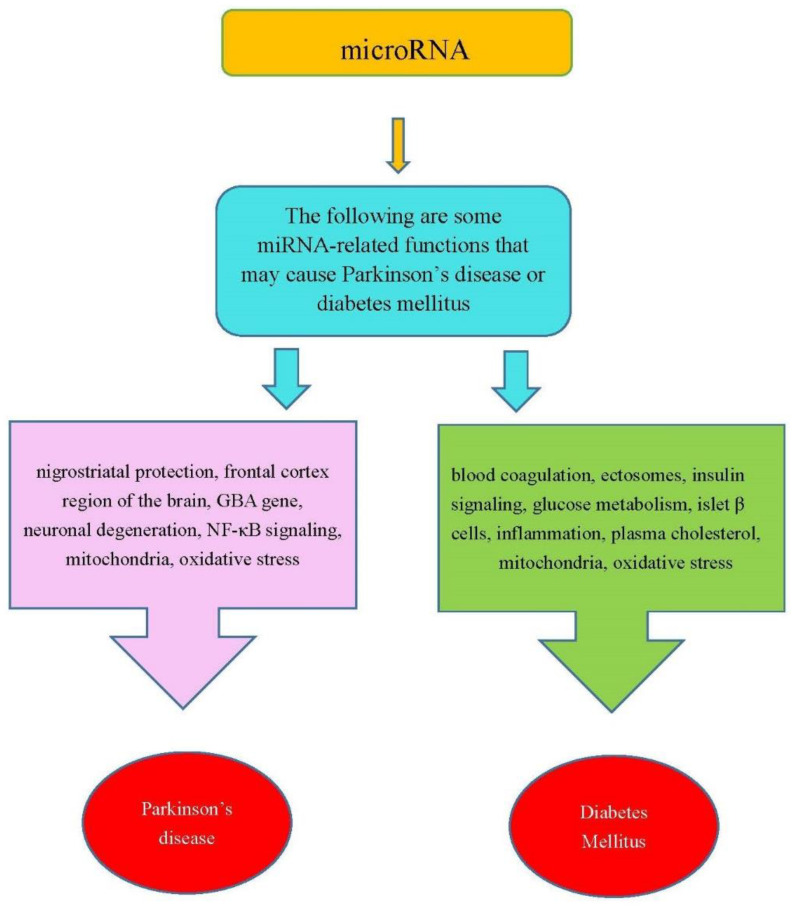
Some miRNA-related functions may cause PD and DM.

In summary, the miRNAs in Table 1 were involved in the functions that are related PD or DM. Figure 1 shows some miRNA-related functions that may cause PD or DM.

## 4. Discussion

In addition to discussing the link of PD and DM through their common miRNA biomarkers, many studies revealed common underlying mechanisms in the pathophysiology of both diseases including hyperglycemia, mitochondrial dysfunction, oxidative stress, and inflammation [186]. PD results mainly from the death of dopaminergic neurons in the substantia nigra. Insulin receptors are relatively plentiful in substantia nigra neurons, and hyperglycemia suppresses substantia nigra dopaminergic neuronal firing. Moreover, insulin resistance has been associated with mitochondrial dysfunction, while MPTP, a toxin that caused numerous cases of parkinsonism, leads to mitochondrial dysfunction [187]. Mitochondria are the key regulator of glucose-stimulated insulin secretion in the pancreatic β cells, and mitochondrial dysfunction was suggested to play a key role in the pathophysiology of DM [188].

Mitochondria contain multiple families of non-coding RNAs including miRNA. miRNAs localizing in mitochondria, known as mitomiRs, were discovered to influence various metabolic pathways. The mitochondrial metabolic pathways are actively involved in energy metabolism during type 2 DM [189]. mitomiRs are also related to neurodegenerative disorders, including Alzheimer’s disease, PD, and Huntington’s disease. MitomiRs play a key role in the pathogenesis of PD by affecting PINK1. Among all neurodegenerative diseases, PD is most affected by mitochondrial dysfunction. MitomiRs related to PD included miR-124, miR-16, miR-21, miR-27a/b, miR-29a, and miR-29b et al. [190]. These miRNAs were involved in the metabolic pathways affected by mitomiRs including TCA, ETC, fatty acid metabolism, or amino acid metabolism pathways, and others. [189].

In addition, the relevance of mitochondrial dysfunction and oxidative stress in the familial and the sporadic forms of PD was explored [191]. Mitochondria represent the major sites for oxidative stress production. Oxidative stress results from an imbalance between harmful reactive oxygen species (ROS) and the antioxidant process that can lead to dopaminergic cell damage in PD as well as pancreatic β cell damage in DM. miRNAs can regulate ROS and prevent ROS-mediated damage to dopaminergic neurons [186]. Studies have focused on how to reduce the damage induced by oxidative stress in both PD and DM. Oxidative stress has been demonstrated as a key factor in the pathogenesis of diabetic nephropathy. Damiano et al. suggested that a red orange and lemon extract could reduce the oxidative damage measured in urine samples in Zucker diabetic fatty rats and prevent renal complications associated with type 2 DM [192,193]. Flavonoids, the phytonutrients present in fruits and vegetables, can provide numerous health benefits and reduce the risk of PD due to their antioxidative, anti-inflammatory, anti-apoptotic, and lipid-lowering properties [194,195].

Moreover, PD and DM also shared the protein aggregation similarity. α-Synuclein, a protein encoded by the SNCA gene, is abundant in the brain and is also localized in neuronal mitochondria [196]. α-Synuclein is a key protein component in the protein inclusions in PD. In familial PD, multiplication and point mutations in the SNCA can lead to toxic aggregation of the α-synuclein protein and subsequent degeneration of dopaminergic neurons in the substantia nigra. Additionally, α-synuclein is also found in the pancreatic β cells. Islet amyloid polypeptide (IAPP) aggregates to form amyloid structures in β cells in type 2 DM. Type 2 DM patients are most likely to develop PD as α-synuclein may combine to amyloid fibrils and form irreversible damaging complexes in dopaminergic cells [197]. An animal study demonstrated increased accumulation, aggregation, and phosphorylation of α-synuclein and IAPP in the pancreatic islets of spontaneously developed type 2 DM monkeys, as compared to the normal subjects. Besides, double immunofluorescence analyses showed a complete overlap between α-synuclein and IAPP in the pancreatic islets [198].

**Figure 2:**
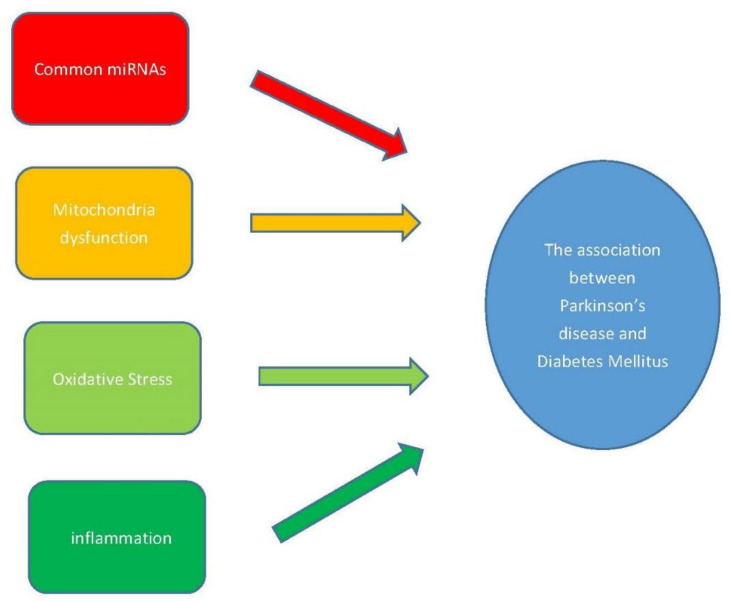
The factors that may contribute to the association of PD and DM.

The aspects from common miRNA biomarkers, and the mechanisms of mitochondrial dysfunction, oxidative stress, and protein aggregation show the connection between PD and DM (Figure 2). In addition to the disease pathology discussion, cohort studies demonstrated the increased risk of PD for DM patients. A comprehensive meta-analysis based on cohort studies in more than 1,761,000 individuals showed that compared to non-diabetic patients, patients with DM were associated with a 38% increase in the risk of developing PD [199]. Another study indicates that DM increased the risk of developing PD in this Chinese population age 20 years and older. The magnitude of association is higher among women and individuals age 65 years and older [200]. In addition, the risk of developing PD in DM patients may depend on the treatment for DM. A large population-based cohort study has shown that the incidence of PD in type 2 DM patients varied substantially depending on the treatment for DM received. The rate of PD was 36–60% lower in users of dipeptidyl peptidase 4 inhibitors and glucagon-like peptide-1 receptor agonists compared to users of other oral anti-diabetic drugs [201].

## 5. Conclusions

miRNAs are shown to be involved in the mechanisms of many diseases including PD and DM. Hence, exploring common miRNA biomarkers of different diseases can help improve our understanding of the relationship of different diseases. In this study, we review common miRNAs that contribute to both PD and DM. Many common miRNAs are discussed in this study. In addition to connecting PD and DM in relation to miRNA, we also discuss their relationships with the mechanisms of mitochondrial dysfunction, oxidative stress, and protein aggregation.

## Data Availability

The study did not report any data.
